# 

**DOI:** 10.1192/bjb.2024.66

**Published:** 2024-12

**Authors:** Tom McWhirter, Ania Korszun

**Affiliations:** Fellow in Medical Education, Wolfson Institute of Population Health, Queen Mary University of London, London, UK; Professor of Psychiatry and Education, Barts and The London School of Medicine and Dentistry, Queen Mary University of London, London, UK; and East London NHS Foundation Trust, London, UK. Email: a.korszun@qmul.ac.uk

**Figure:**
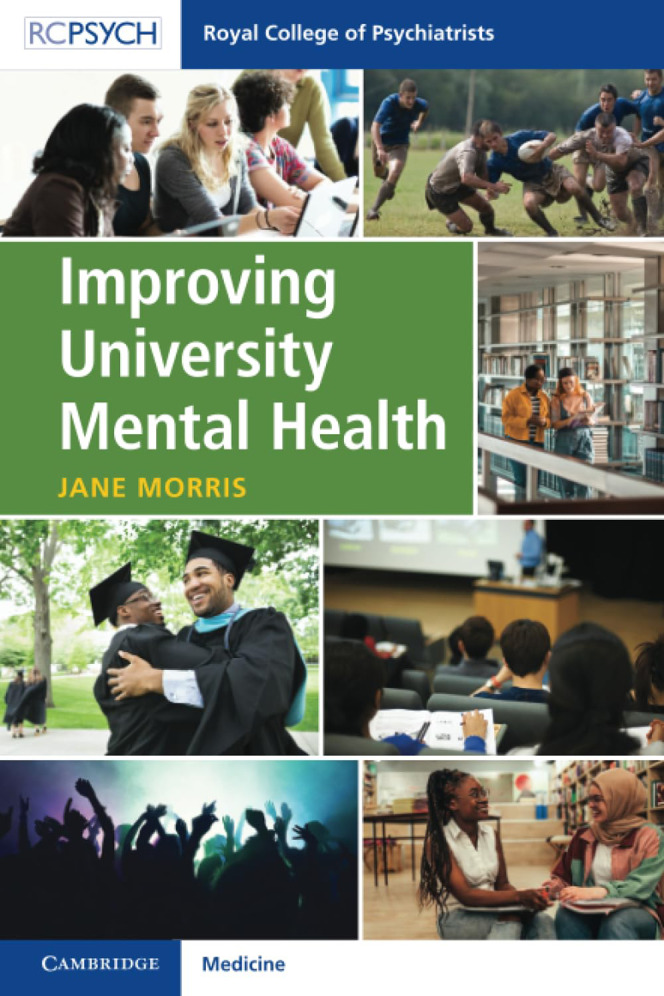

Jane Morris set herself an ambitious goal in addressing all aspects of improving university mental health, which range from choice of university to facing the myriad challenges of university life. This book is an excellent guide to the current state of UK university mental health and she has certainly met her goal.

The book has 20 chapters, which can be read in isolation. It also provides clear signposts linking related topics across the book. The prose is clear, with jargon clarified, making it accessible to parents, faculty and students, as well as healthcare professionals. There are case vignettes that highlight specific difficulties and nicely illustrate a range of outcomes. Each chapter comes with a useful summary of ‘practice points’, elucidating where changes might be made. There are clearly laid-out references.

The author goes beyond thinking about students in isolation and considers the health of the whole system in which young people make their transition to higher education. A range of mental health conditions is covered, as well as factors such as ethnicity, sexuality, alcohol, social media and finances and how these intersect with student mental health. There are good summaries to ground those unfamiliar with the area, and one of the recurring messages of this book is that no one lives with mental illness in a vacuum. The continual focus on social structures and relationships as key to thriving mental health is valuable.

Importantly, this book has been written by a psychiatrist and it gives a confident approach to diagnosable illness and mental wellbeing, with the distinction between the two made clear. Thoughtful positions are taken on the utility and drawbacks of diagnosis, on the place of medical treatment and the considerations needed for both in the case of young and developing students. This makes it especially helpful for non-medical university services and for parents who wish to develop a common language and understanding with the medical mental health services looking after their children.

The author calls for accessible, compassionate and communicative networks. She makes a compelling case for improved interaction across mental health services and of harnessing the expertise of psychiatrists, including Child and Adolescent Mental Health Services (CAMHS) clinicians. She suggests having a Faculty of University Mental Health at the Royal College of Psychiatrists (which the reviewers would certainly want to join).

We can highly recommend this book as an invaluable guide for anyone who is interested in improving university mental health.

